# Rapid Surface Modification of Ultrafiltration Membranes for Enhanced Antifouling Properties

**DOI:** 10.3390/membranes10120401

**Published:** 2020-12-07

**Authors:** Noresah Said, Ying Siew Khoo, Woei Jye Lau, Mehmet Gürsoy, Mustafa Karaman, Teo Ming Ting, Ebrahim Abouzari-Lotf, Ahmad Fauzi Ismail

**Affiliations:** 1 Advanced Membrane Technology Research Centre (AMTEC), School of Chemical and Energy Engineering, Universiti Teknologi Malaysia, Skudai 81310, Malaysia; noresahsaid@yahoo.com (N.S.); yingsiew520@gmail.com (Y.S.K.); afauzi@utm.my (A.F.I.); 2 Department of Chemical Engineering, Konya Technical University, Konya 42075, Turkey; mgursoy@ktun.edu.tr (M.G.); mkaraman@ktun.edu.tr (M.K.); 3 Radiation Processing Technology Division, Malaysian Nuclear Agency, Kajang 43000, Malaysia; tmting@nm.gov.my; 4 Helmholtz Institute Ulm (HIU) Electrochemical Energy Storage, Helmholtzstraße 11, D-89081 Ulm, Germany

**Keywords:** ultrafiltration, membrane, PECVD, hydrophilicity, antifouling property, protein purification

## Abstract

In this work, several ultrafiltration (UF) membranes with enhanced antifouling properties were fabricated using a rapid and green surface modification method that was based on the plasma-enhanced chemical vapor deposition (PECVD). Two types of hydrophilic monomers—acrylic acid (AA) and 2-hydroxyethyl methacrylate (HEMA) were, respectively, deposited on the surface of a commercial UF membrane and the effects of plasma deposition time (i.e., 15 s, 30 s, 60 s, and 90 s) on the surface properties of the membrane were investigated. The modified membranes were then subjected to filtration using 2000 mg/L pepsin and bovine serum albumin (BSA) solutions as feed. Microscopic and spectroscopic analyses confirmed the successful deposition of AA and HEMA on the membrane surface and the decrease in water contact angle with increasing plasma deposition time strongly indicated the increase in surface hydrophilicity due to the considerable enrichment of the hydrophilic segment of AA and HEMA on the membrane surface. However, a prolonged plasma deposition time (>15 s) should be avoided as it led to the formation of a thicker coating layer that significantly reduced the membrane pure water flux with no significant change in the solute rejection rate. Upon 15-s plasma deposition, the AA-modified membrane recorded the pepsin and BSA rejections of 83.9% and 97.5%, respectively, while the HEMA-modified membrane rejected at least 98.5% for both pepsin and BSA. Compared to the control membrane, the AA-modified and HEMA-modified membranes also showed a lower degree of flux decline and better flux recovery rate (>90%), suggesting that the membrane antifouling properties were improved and most of the fouling was reversible and could be removed via simple water cleaning process. We demonstrated in this work that the PECVD technique is a promising surface modification method that could be employed to rapidly improve membrane surface hydrophilicity (15 s) for the enhanced protein purification process without using any organic solvent during the plasma modification process.

## 1. Introduction

For the past several decades, ultrafiltration (UF) membrane technology was widely used for the separation of macromolecules from the aqueous solution for a wide range of industrial applications [1,2,3]. One important application of UF membranes is to provide high retentions of protein, involving the separation of biological solution comprised of amino acid, peptides and enzymes [4]. Nevertheless, the major concern of using UF membranes for protein separation is the deterioration of water productivity as a result of membrane surface fouling caused by the protein deposition/adsorption which leads to an increase in water transport resistance [2]. 

The commercially available UF membranes are mainly made of polymeric materials (e.g., polysulfone (PSf), polyethersulfone (PES), and polyvinylidene difluoride (PVDF)) that are not hydrophilic in nature [5,6,7]. There are many studies which have reported the low antifouling properties of these UF membranes during the filtration process. For instance, Jia et al. [8] found the severe flux decline (>80% flux reduction) of the PSf-based membrane after being tested for 1000 mg/L bovine serum albumin (BSA) solution for 5 h. Jamshidi Gohari et al. [9] reported that the flat sheet membrane made of PES suffered from great irreversible fouling resistance (R_ir_: 0.46) compared to the nanomaterials-modified PES membrane (R_ir_: 0.04) after both membranes were tested under the same conditions using 1000 mg/L BSA solution for 2 h. Rahimi et al. [10] on the other hand reported that PVDF membrane was very susceptible to the organic foulants present in the solution. The statement was supported by the remarkable flux deterioration (>66% flux reduction) and the formation of a cake layer on the membrane surface. 

Two main strategies to improve the membrane antifouling properties are (1) bulk membrane modification via incorporation of hydrophilic nanomaterials [11,12,13,14] or hydrophilic polymers [15,16,17] and (2) top surface modification using methods such as dip-coating [18,19] and layer-by-layer (LbL) assembly [19,20,21]. Nevertheless, these surface modification approaches are always associated with significant drawbacks. One of them is the requirement of using large quantity of inorganic nanoparticles, i.e., as high as 3–4 wt% in the dope formulation [22,23]. Although the membrane surface hydrophilicity was able to improve upon the nanomaterial incorporation, the resultant membrane morphology is usually found to be adversely affected (e.g., enlarged pore size and/or poor mechanical stability) owing to the uneven distribution of the nanoparticle and/or its severe aggregation [24,25]. In addition, the possible leaching of nanoparticles from the membrane matrix is another main concern of using this kind of membrane. 

In general, most of the top surface modification approaches are labor-intensive which require long working hours to achieve desired properties. For instance, Tran et al. [26] reported that up to 26 h was needed to complete the coating process of the polydopamine/amine-terminated polysiloxane (PDA/PSI-NH_2_) on the PSf membrane surface. Although Bai et al. [27] utilized a relatively short modification period (30 min) to coat cellulose-based materials on the PES membrane using an ultrasonic treatment, the effective membrane coating area was very low, i.e., 50 cm^2^. It must be pointed out that most of the modification approaches are not environmentally friendly as organic solvents/chemicals are generally needed for nanoparticle surface functionalization and/or membrane post-treatment. Although Mitev et al. [28] employed a solvent-free plasma-enhanced chemical vapor deposition (PECVD) to modify the surface of polyetherimide (PEI)-based membrane using hexamethyldisiloxane (HS) as a monomer, the modified membranes were only used for the organic solvent separation process.

In this work, we intend to use a solvent-free PECVD method to rapidly modify the surface properties of the UF membrane to achieve desirable fouling resistance during the water treatment process. During the PECVD process, the plasma polymerization of the precursors, which is induced in the plasma stream, is of the random radical recombination type. For this reason, there is always a radical that initiates the process of polymerization and makes it so fast to form the unique and extremely thin layer [29]. 

The primary objective of this study is to develop a highly antifouling resistant UF membrane using a rapid and green PECVD method to handle feed solution containing a high concentration of foulants. Two hydrophilic monomers which are acrylic acid (AA) and 2-hydroxyethyl methacrylate (HEMA) were, respectively, used to modify the surface properties of the UF membrane, and the impacts of plasma deposition duration (ranging from 15 s to 90 s) on the physiochemical properties of the UF membrane were then evaluated using a series of analytical instruments prior to filtration process using BSA and pepsin as the model proteins. 

## 2. Materials and Methods 

### 2.1. Materials

**Figure 1:**
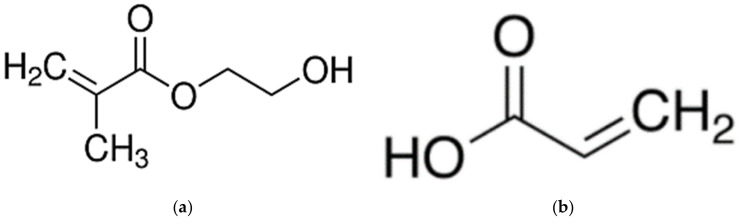
Chemical structure of (**a**) 2-hydroxyethyl methacrylate (HEMA) (MW: 130.14 g/mol) and (**b**) acrylic acid (AA) (MW: 72.06 g/mol).

A commercial flat sheet UF membrane (PS20, molecular weight cut-off: 20 kDa) supplied in dry condition by RisingSun Membrane Technology (Beijing, China) Co. Ltd. was utilized in the work as the substrate for PECVD surface modification. Two monomers, i.e., 2-hydroxyethyl methacrylate (HEMA, 97%) and acrylic acid (AA, 99%) purchased from Sigma-Aldrich (Darmstadt, Germany) were used without further purification to modify the surface characteristics of the UF membrane. The chemical structures of these two hydrophilic monomers are shown in Figure 1. Bovine serum albumin (BSA, 66.5 kDa) and pepsin from porcine gastric mucosa (35 kDa) purchased from Sigma-Aldrich were used to determine membrane rejection and antifouling behavior by dissolving respective solutes in pure water. 

### 2.2. Surface Modification of the UF Membrane

**Figure 2:**
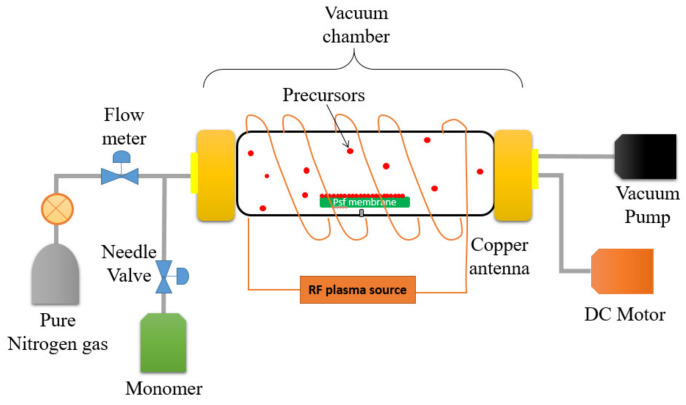
Illustration of the plasma-enhanced chemical vapor deposition (PECVD) process for surface modification of a polysulfone (PSf) flat sheet membrane.

A self-customized PECVD system as illustrated in Figure 2 was utilized to modify the surface properties of the UF membrane by depositing two different monomers onto the membrane surface under vacuum conditions. A cylindrical quartz vacuum chamber 30-cm in length and 6-cm in diameter was utilized as a reactor. The UF membrane sample (Dimension: 6 cm × 15 cm) was then placed on flat silicon wafers before being transferred to the chamber. A copper coil antenna connected with 13.56 MHz radiofrequency plasma generator was surrounded through the quartz window of the chamber. First and foremost, a vacuum condition was created within the chamber by switching on the vacuum pump with the aim to eliminate air within the reactor. The monomer (either AA or HEMA) was then vaporized in a stainless-steel jar before it was delivered to the vacuum chamber by controlling the fine metering valve. A backside cooling plate was installed as a substrate holder in order to maintain the membrane temperature. The desired operating pressure (<100 mtorr) in the chamber was maintained using a proportional integral derivative (PID) controlled butterfly valve which connected to a capacitance manometer. In this work, the optimized flow rate and plasma power of AA (0.75 sccm; 40 W) and HEMA deposition (0.4 sccm; 50 W) were used throughout the modification process. The only variable during PECVD process was the plasma deposition time. For each monomer, the impacts of its deposition time (i.e., 15 s, 30 s, 60 s, and 90 s) on the membrane surface properties were investigated. These resultant membranes samples are then denoted as AA-modified membranes and HEMA-modified membranes, depending on the type of monomer deposited on the membrane surface.

### 2.3. Characterization of Surface-Modified UF Membranes

The chemical composition of the membrane surface with and without plasma modification was determined using Fourier transmission Infrared (FTIR) spectrometer (Nicolet 5700, Thermo Scientific, Madison, WI, USA) with wavenumber ranging from 600 to 4000 cm^−1^. All the membrane samples were dried in an oven at 35 °C for 24 h prior to FTIR analysis. The surface wettability (hydrophilicity) of membranes was evaluated using a contact angle goniometer (OCA 15Pro, DataPhysics Instruments, Filderstadt, Germany) via the sessile drop method. At least 10 measurements were randomly performed on the membrane sample to yield the average result. A field emission scanning electron microscope (FESEM) (Crossbeam 340, ZEISS, Oberkochen, Germany) was used to compare the surface morphology and cross-section of the membrane with and without plasma modification. All the membrane samples were sputter coated with platinum in order to avoid the surface charging effect. X-ray photoelectron spectroscopy (XPS) analysis was performed on selected membrane samples using X-ray photoelectron spectrometer (K-Alpha, Thermo Scientific, Hong Kong, China) to gain an in-depth understanding of the membrane surface chemical elemental composition.

### 2.4. Membrane Performance Evaluation

The pure water permeability of membranes was determined in a dead-end mode using a stainless-steel permeation cell (HP4750, Sterlitech Corp., Kent, OH, USA). Each membrane sample with an active surface area of 14.6 cm^2^ was first compressed at 2 bar using reverse osmosis (RO) water for 30 min to achieve flux stability. Then, the membrane was evaluated with respect to its pure water permeability at 1 bar. At least three measurements were performed on the same membrane sample to yield an average value. The membrane pure water permeability, J (L/m^2^ h bar) was evaluated based on the following equation:

(1) $$J=\frac{∆V}{A\times ∆t\;\times \Delta P}$$

where Δ*V*, *A*, Δ*t* and ΔP represent the volume of permeate obtained (L), membrane active surface area (m^2^), time for collecting permeate volume (h), and operating pressure (bar), respectively.

After completing the pure water permeability test, the membrane samples were evaluated for their rejection capability against BSA and pepsin. The rejection test was conducted at 1 bar using either 2000 mg/L BSA solution or 2000 mg/L pepsin solution. The membrane rejection, *R* (%) was evaluated using:

(2) $$R\;\left(\%\right)=\;\left(1-\frac{C_{p}}{C_{F}}\right)\;\times 100$$

where $C_{F}$ is the solute concentration (mg/L) in feed solution and $C_{p}$ is the solute concentration (mg/L) in permeate solution.

### 2.5. Membrane Fouling Test

The antifouling test was further carried out to examine the fouling resistance of modified membranes against organic foulant. An amount of 2000 mg/L of BSA solution was used as a feed solution to investigate the fouling behavior of membranes for a duration of 4 h at an operating pressure of 1 bar. The permeate flux was collected every 30 min and a graph of normalized permeability (P/P_o_) against filtration duration was then plotted to analyze the flux behavior of membrane, whereby P_o_ represented the initial permeate flux of membrane while P is the final permeate flux during filtration. In order to maintain the properties of feed solution throughout the operation, the permeate collected was recycled to the feed after it was analyzed. After completing the fouling test, the BSA feed solution was replaced with pure water and the fouled membrane was surface-cleaned with the water for 15 min at a stirring speed of 350 rpm without applying any external force. After that, the pure water permeability of the surface-cleaned membrane was re-measured in order to determine its flux recovery rate (FRR, %) using the following equation.

(3) $$\mathrm{FRR}\;=\;\frac{J_{2}}{J_{1}}\times \;100\;$$

where *J*_2_ is the pure water flux of membrane after water cleaning while *J*_1_ is the initial water flux of pristine membrane. 

## 3. Results and Discussion

### 3.1. Membrane Surface Chemistry

**Figure 3:**
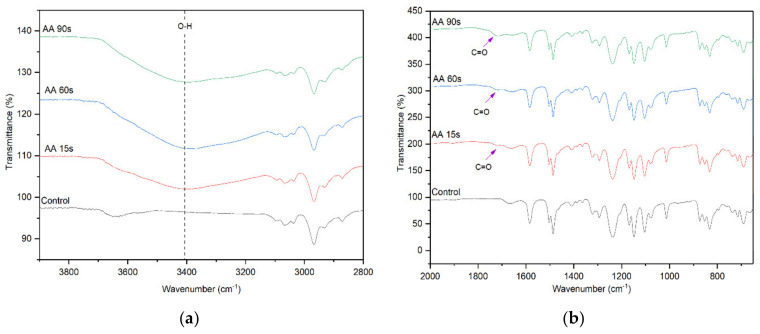
FTIR spectra of the control membrane and AA-modified membranes, (**a**) wavenumber of 3800–2800 cm^−1^ and (**b**) wavenumber of 2000–600 cm^−1^.

The FTIR spectra of control and AA-modified membranes at various plasma deposition times are presented in Figure 3. The peak intensity of the –OH functional group at 3400 cm^−1^ for the AA-modified membrane is significantly higher than that of the unmodified control membrane, indicating the successful deposition of AA onto the membrane surface. The increase in the peak intensity for the AA-modified membrane with increasing plasma deposition time from 15 s to 90 s signifies the increased number of –OH functional groups originated from the chemical structure of AA [30]. Other distinctive surface chemistry between the control membrane and the AA-modified membranes is found at 1710 cm^−1^. This peak is attributed to the C=O stretching vibration of AA. These findings provide a clear indication of the successful deposition of AA on the surface of the membrane. 

**Figure 4:**
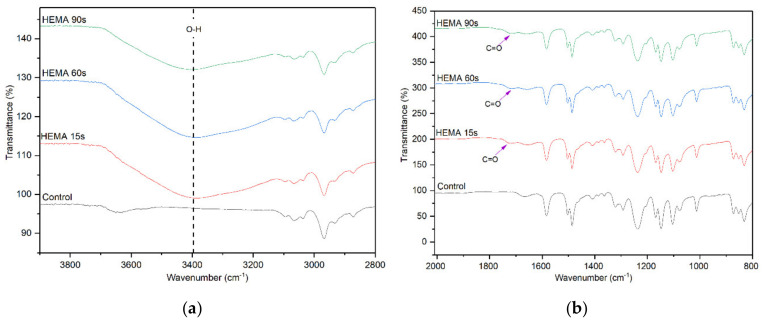
FTIR spectra of control membrane and HEMA-modified membranes, (**a**) wavenumber of 3800–2800 cm^−1^ and (**b**) wavenumber of 2000–800 cm^−1^.

The effect of HEMA deposition time on the surface chemistry of the membranes was also evaluated and the FTIR results are shown in Figure 4. Similar to the AA deposition, the membranes with HEMA deposition also show a significantly higher peak intensity at 3400 cm^−1^. However, compared to the AA-modified membranes, the –OH peak intensity of HEMA-modified membranes does not increase obviously with increasing plasma deposition time from 15 to 90 s. Furthermore, the peak attributed to C=O stretching vibration as a result of HEMA deposition can also be observed at 1710 cm^−1^ for all the HEMA-modified membranes, confirming the presence of HEMA on the membrane surface. The finding is in agreement with Hu et al. [31] in which HEMA was used to modify the surface of commercial polypropylene microporous membranes via grafting approach.

### 3.2. Membrane Surface Chemistry

**Figure 5:**
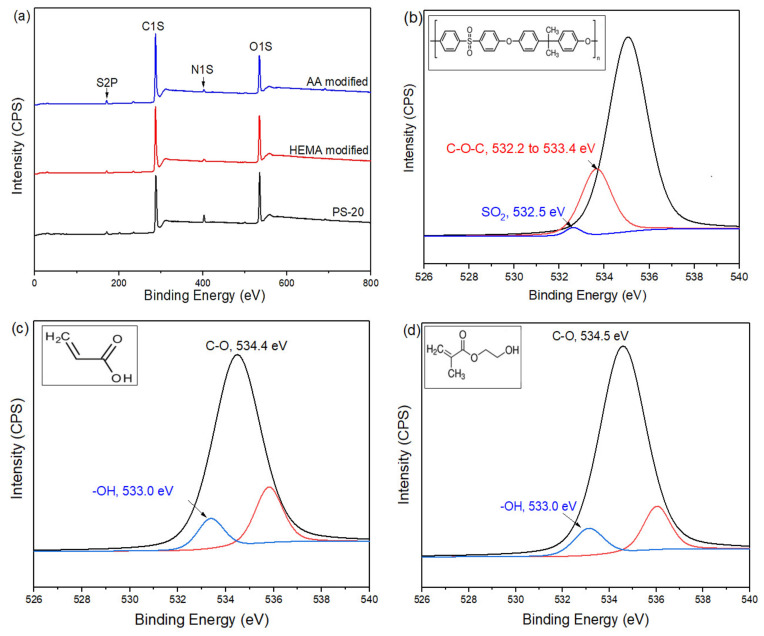
XPS wide scans of (**a**) the control membrane (PS-20) and modified membranes and high-resolution O1s spectra of (**b**) the control (Inset: Organic structure of PSf), (**c**) AA-modified (inset: organic structure of AA), and (**d**) HEMA-modified membranes (inset: organic structure of HEMA).

The chemical composition of the membranes with and without plasma modification was further analyzed via XPS and the results are shown in Figure 5. As can be clearly seen, all of the membranes possess the same peaks at 530, 400, 285, and 164 eV, regardless of surface modification type. These peaks correspond to binding energies of oxygen, nitrogen, carbon, and sulfur, respectively. The existence of these elements is due to the organic polymers used in membrane making/surface modification. Further analysis based on the high-resolution spectrum of O1s strongly suggests the existence of AA or HEMA on the membrane surface. The O1s peak of AA-modified membrane can be curve-fitted into two components with binding energies at about 533.0 eV and 534.4 eV which are attributed to –OH and C–O bonds [32,33], respectively. This confirms the formation of polyacrylic acid (PAA) on the control membrane surface. Meanwhile, the high resolution O1s peak of HEMA modified membrane can be also deconvoluted into two different peaks, i.e., 533.0 eV and 534.5 eV. Both peaks are ascribed to –OH and C–O/C–OH bonds [34], respectively. These significant characteristic peaks confirm the presence of a polyhydroxyethyl methacrylate (PHEMA) coating layer atop the control membrane. It is also worth noting that the O1s spectrum of control membrane is only dominated by two main peaks at 532.3 eV and 533.3 eV which belong to C–O–C and S=O bonds, respectively [35,36]. 

### 3.3. Membrane Surface Hydrophilicity

**Figure 6:**
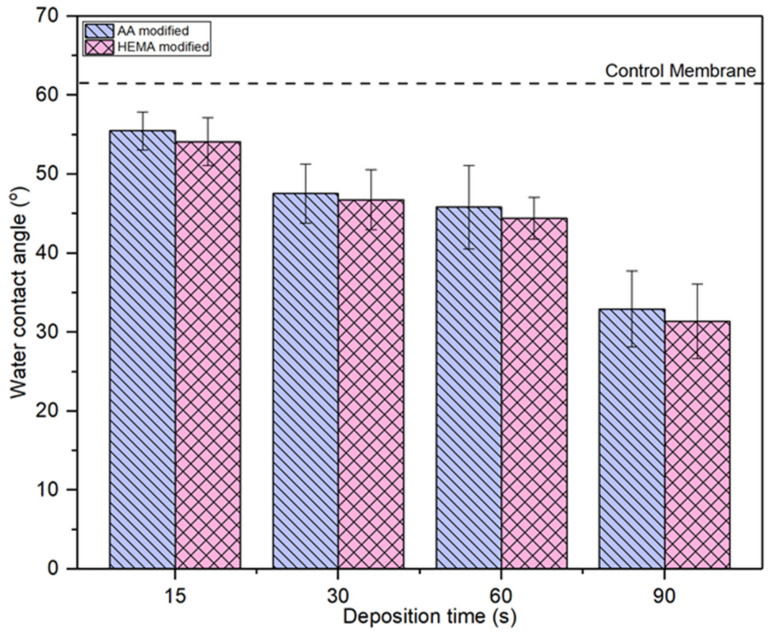
Comparison of surface water contact angle of membranes modified by different monomers at different deposition time. Error bars indicate standard deviations.

Figure 6 compares the water contact angle of AA- and HEMA-modified membranes at various plasma deposition times. It can be seen that all the modified membranes show lower water contact angle compared to the control membrane, indicating the improved membrane surface hydrophilicity upon the plasma modification. For both types of modified membranes, the trend of water contact angle is exactly the same, i.e., the higher the plasma modification duration the lower the membrane water contact angle or vice versa. The water contact angle for the AA-modified membrane and HEMA-modified membrane decreases gradually from 55.48° to 32.94° and from 55.97° to 43.36°, respectively, with increasing plasma deposition duration from 15 s to 90 s. The decreasing water contact angle is a strong indication of the improved membrane surface hydrophilicity. This statement is also supported by the high peak intensity of –OH groups at the modified membranes as presented in Figure 3 and Figure 4. This allows the modified membranes to have higher affinity towards water molecules [37]. The membrane with a higher degree of hydrophilicity is important for the water treatment process as it can mitigate fouling caused by protein deposition/adsorption [38]. 

### 3.4. Membrane Morphology

**Figure 7:**
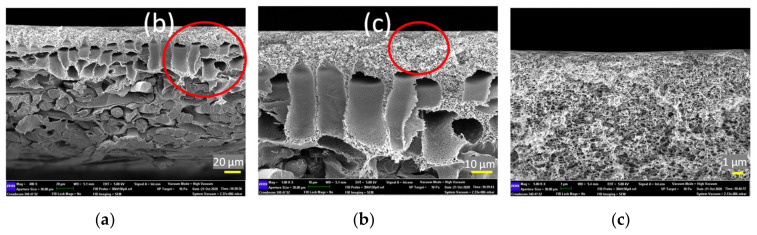
Cross-section structure of control membrane at different scale bars, (**a**) 20 μm, (**b**) 10 μm, and (**c**) 1 μm (the red circles in the photographs indicate the enlarged area for further analysis).

Figure 7 shows the cross-sectional morphology of the control membrane (a) at different magnifications (b and c). This membrane is commercially available and is fabricated via the phase inversion method by establishing an asymmetric microporous membrane over a non-woven fabric. From the FESEM images, it can be seen that this membrane is composed of three distinct layers, i.e., a selective skin layer on top of a macrovoid sub layer supported by a thick fabric. The selective skin layer is the main important part of the asymmetric membrane as it governs the solute rejection rate as well as water productivity rate. 

**Figure 8:**
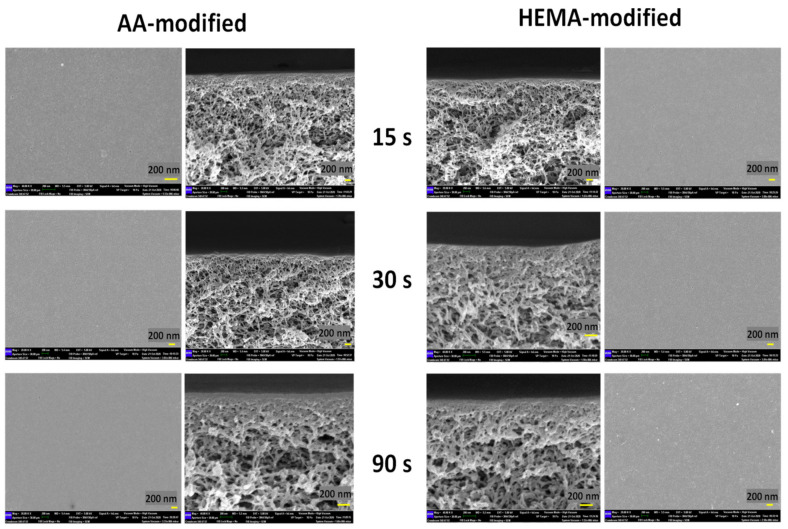
Surfaces and cross-sections of AA-modified and HEMA-modified membranes at various plasma deposition times of 15 s, 30 s, and 90 s.

The impacts of plasma modification on the membrane structure were further investigated and the FESEM images as shown in Figure 8 indicate that there is no significant difference on the surface and cross-sectional morphology of the modified membranes, except a thicker selective layer (at nm-scale) is found on the membranes modified by the highest plasma deposition duration (90 s). Such a finding is interesting but in good agreement with our previous studies in which the PECVD technique was utilized to develop an extremely thin layer on the surface of thin film composite nanofiltration and reverse osmosis membranes [37] and polymeric foams [39]. 

### 3.5. Membrane Pure Water Flux and Rejection

**Figure 9:**
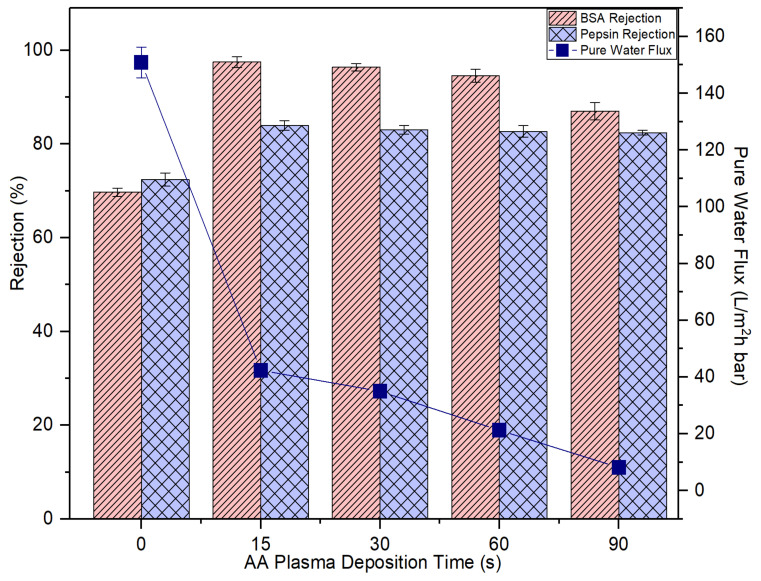
The impact of plasma deposition time of AA on the membrane pure water flux and solute rejection using 2000 mg/L bovine serum albumin (BSA) solution and 2000 mg/L pepsin solution. Error bars indicate standard deviations.

Figure 9 presents the effects of AA deposition time on the membrane pure water flux and solute rejection. By increasing the plasma deposition time, it is found that the membrane pure water flux is negatively affected. This result can be explained by the fact of the increased membrane transport resistance as a result of the presence of a thin but dense coating layer atop its surface as evidenced by the FESEM images (Figure 8). The increasing AA layer thickness due to the increased plasma deposition time has increased the transport resistance for water molecules to pass through the membrane, thus affecting the membrane pure water flux [40]. 

However, it must be pointed out the membrane rejections against BSA (67 kDa) and pepsin (36 kDa) are improved upon plasma modification. At 15-s plasma deposition, the resultant modified membrane could improve the BSA and pepsin rejection from 69.7% to 97.5% and from 72.4% to 83.9%, respectively. That is, 27.8% and 11.5% enhancement compared to the control membrane, respectively. The results are due to decrease in the membrane pore size which corresponds to the reduced membrane water flux. The increased coating layer thickness is closely linked to the narrowing or blockage of the pores. Further increase in the plasma deposition time from 15 s to 90 s, however, did not have a positive effect on solute rejection. This is mainly because the plasma layer deposited is not able to form the extremely small pore size that can completely eliminate BSA and pepsin, although its coating layer thickness is increased with increasing plasma deposition time.

**Figure 10:**
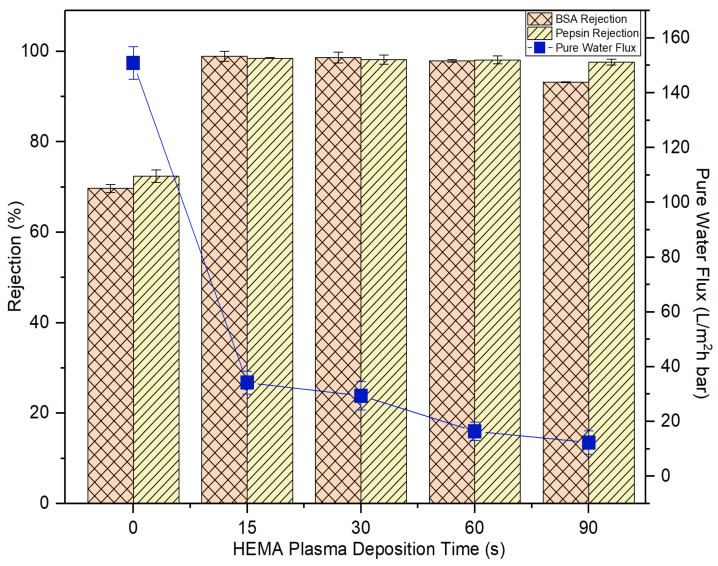
The impact of plasma deposition time of HEMA on the membrane pure water flux and solute rejection using 2000 mg/L BSA solution and 2000 mg/L pepsin solution. Error bars indicate standard deviations.

Concerning the HEMA deposition on the membrane, it can be found that its impacts on the membrane pure water flux and protein rejection are very similar to the effects imposed by AA deposition. Figure 10 also shows that the water flux of the HEMA-modified membranes is reduced with increasing plasma deposition time and the membrane rejection remains similar after 15-s plasma modification. For both sets of experimental work, it can be said that the 15-s plasma modification duration is the best condition to produce modified membranes with the highest BSA and pepsin rejection without having to experience a further drop in the water flux. In comparison, the 15-s HEMA-modified membrane displays slightly better pure water flux and protein rejection than the 15-s AA-modified membrane. While AA has a carboxyl group in its molecular structure, HEMA has methyl, hydroxyl and ester bonds [41]. The functional hydroxyl group of HEMA tends to make the HEMA-modified membrane more reactive to attract water molecules, leaving the protein molecules behind. In the following section, only the membranes modified at 15-s deposition time were selected for further investigation to compare their antifouling properties with the control membrane. 

### 3.6. Membrane Antifouling Properties

**Figure 11:**
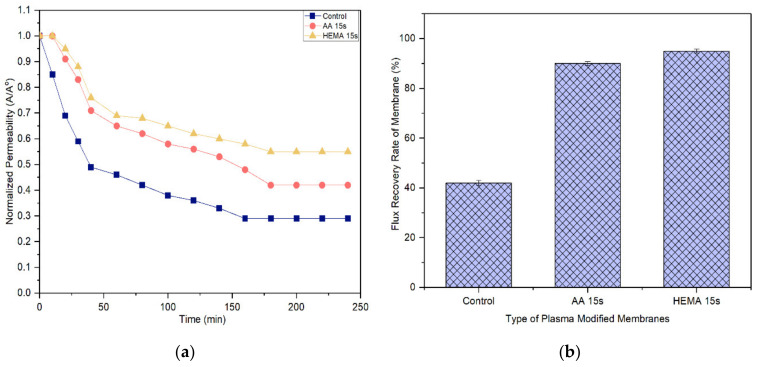
(**a**) Flux decline curves of membranes using 2000 mg/L BSA solution and (**b**) flux recovery rate (FRR) of membranes after water cleaning. Error bars indicate standard deviations.

**Figure 12:**
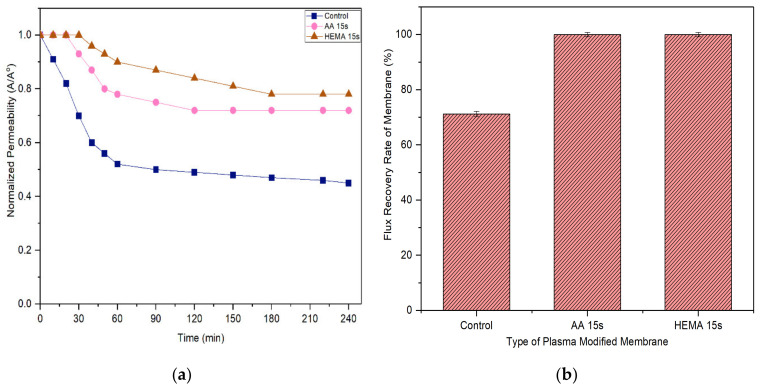
(**a**) Flux decline curves of membranes tested with 2000 mg/L pepsin solution and (**b**) flux recovery rate (FRR) of membranes after water cleaning. Error bars indicate standard deviations.

A membrane with less tendency to foul is more favorable in the industrial process as it can last longer before a cleaning process is carried out. Figure 11 and Figure 12 compare the normalized permeability and FRR of modified membranes with the control membrane in filtrating feed solution containing different foulants. For both model proteins, the curves representing the control, AA-modified and HEMA-modified membranes can be divided into three distinct phases during fouling. The first phase, which is rapid flux decline, occurs in the early stage of filtration (<50 min). During this period, the control membrane experiences a higher degree of flux reduction compared to the modified membranes. The severe fouling of the control membrane is mainly due to its low degree of surface hydrophilicity that favors the adsorption of foulants onto its surface, causing greater concentration polarization. The second phase of fouling, which happens at a moderate rate, is due to the protein deposition on the membrane surface. Eventually, the curves flatten at the third phase of fouling, where the time taken to achieve the constant normalized permeability can indicate the membrane antifouling performance. The constant normalized permeability indicates that a further filtration process would not render obvious flux deterioration. At this stage, the fouling layer caused by the retained proteins would be established on the membrane surface.

Based on the experimental data, it can be found that the AA-modified and HEMA-modified membranes take an average of 180 min for the BSA and 210 min for the pepsin to reach the steady flux. In other word, the membranes experience a lower degree of fouling during pepsin solution filtration. As the pepsin (35 kDa) has a relatively smaller molecular size compared to the BSA (66.5 kDa), it is less likely to cause membrane pores blockage/narrowing than the BSA. The statement is supported by its lower removal rate compared to the BSA rejection (Figure 9 and Figure 10). Another possible factor can be due to the higher adsorption affinity of BSA than that of pepsin, causing it to foul the membrane at a faster rate.

It must be noted that the enhanced surface hydrophilicity of the modified membrane has obviously increased the control membrane’s antifouling properties, minimizing the flux deterioration as a function of time. The hydration layer on the modified membrane surface could slow down the hydrophobic protein from depositing onto the pores and thus reduce the fouling propensity [41,42,43].

With respect to FRR, the AA-modified and HEMA-modified membranes show much higher values compared to the control membrane for both the filtration processes of BSA and pepsin. This strongly indicates the reversible fouling of the modified membranes. The modified membranes achieve almost complete FRR (i.e., 100%) in the pepsin filtration and slightly lower FRR (90–95%) in the BSA filtration. The control membrane meanwhile only shows 71.2% and 42% FRR, respectively, when tested under the same conditions. The results are promising as most of the foulants can be reversibly removed with the use of only pure water (instead of chemical cleaning agents), revealing the positive features of enhanced membrane surface hydrophilicity to induce a more stable selective layer for better antifouling properties [44]. 

## 4. Conclusions

A rapid and green technology based PECVD technique was successfully employed to modify the surface properties of a UF membrane to enhance its fouling resistance in handling feed solution composed of high concentration of proteins. Two types of hydrophilic materials, i.e., AA and HEMA were deposited on the top surface of the membrane and their respective plasma deposition duration was evaluated with respect to the membrane surface physiochemical properties and filtration performance. The water contact angle analysis supported by XPS and FTIR indicated significant enhancement of membrane surface hydrophilicity due to the deposition of AA and HEMA on the membrane surface. Based on the FESEM images, it can be found that the coating layer became thicker with increasing plasma deposition time and the increase in the coating layer thicker corresponded well with the decrease in membrane water flux. The study revealed that the ideal plasma deposition time for AA and HEMA on the membrane was only 15 s. These two surface-modified membranes also demonstrated much higher FRR values compared to the control membrane, indicating that the fouling is mainly reversible and could be easily removed via a simple water cleaning process. Our findings also indicated that the HEMA-modified membrane achieved a lower flux decline rate and higher FRR compared to the AA-modified membrane but possessed slightly lower pure water flux. The discussed PECVD technique is a promising surface modification method to rapidly improve membrane surface hydrophilicity for enhanced protein purification process without using any organic solvent during the plasma process. However, the conditions of PECVD process need to be further optimized in order to minimize the flux decline rate of the modified membranes.
